# Critical Transition in Tissue Homeostasis Accompanies Murine Lung Senescence

**DOI:** 10.1371/journal.pone.0020712

**Published:** 2011-06-21

**Authors:** Carla L. Calvi, Megan Podowski, Franco R. D'Alessio, Shana L. Metzger, Kaori Misono, Hataya Poonyagariyagorn, Armando Lopez-Mercado, Therese Ku, Thomas Lauer, Christopher Cheadle, C. Conover Talbot, Chunfa Jie, Sharon McGrath-Morrow, Landon S. King, Jeremy Walston, Enid R. Neptune

**Affiliations:** 1 Division of Pulmonary and Critical Care Medicine, Johns Hopkins University School of Medicine, Baltimore, Maryland, United States of America; 2 Pediatric Pulmonary, Johns Hopkins University School of Medicine, Baltimore, Maryland, United States of America; 3 Lowe Family Genomics Core, Division of Allergy and Clinical Immunology, Johns Hopkins University School of Medicine, Baltimore, Maryland, United States of America; 4 JHMI Microarray Core, Johns Hopkins University School of Medicine, Baltimore, Maryland, United States of America; 5 Division of Geriatrics, Johns Hopkins University School of Medicine, Baltimore, Maryland, United States of America; University of Giessen Lung Center, Germany

## Abstract

**Background:**

Respiratory dysfunction is a major contributor to morbidity and mortality in aged populations. The susceptibility to pulmonary insults is attributed to “low pulmonary reserve”, ostensibly reflecting a combination of age-related musculoskeletal, immunologic and intrinsic pulmonary dysfunction.

**Methods/Principal Findings:**

Using a murine model of the aging lung, senescent DBA/2 mice, we correlated a longitudinal survey of airspace size and injury measures with a transcriptome from the aging lung at 2, 4, 8, 12, 16 and 20 months of age. Morphometric analysis demonstrated a nonlinear pattern of airspace caliber enlargement with a critical transition occurring between 8 and 12 months of age marked by an initial increase in oxidative stress, cell death and elastase activation which is soon followed by inflammatory cell infiltration, immune complex deposition and the onset of airspace enlargement. The temporally correlative transcriptome showed exuberant induction of immunoglobulin genes coincident with airspace enlargement. Immunohistochemistry, ELISA analysis and flow cytometry demonstrated increased immunoglobulin deposition in the lung associated with a contemporaneous increase in activated B-cells expressing high levels of TLR4 (toll receptor 4) and CD86 and macrophages during midlife. These midlife changes culminate in progressive airspace enlargement during late life stages.

**Conclusion/Significance:**

Our findings establish that a tissue-specific aging program is evident during a presenescent interval which involves early oxidative stress, cell death and elastase activation, followed by B lymphocyte and macrophage expansion/activation. This sequence heralds the progression to overt airspace enlargement in the aged lung. These signature events, during middle age, indicate that early stages of the aging immune system may have important correlates in the maintenance of tissue morphology. We further show that time-course analyses of aging models, when informed by structural surveys, can reveal nonintuitive signatures of organ-specific aging pathology.

## Introduction

A stereotyped pattern of structural changes which occur in the human lung as it ages, termed “senile lung”, is characterized by airspace enlargement that is similar but not identical to acquired emphysema [1], [2]. Although the chronicity of this process is poorly understood with respect to time of onset or progression, the reproducibility of the underlying pattern suggests that the lung harbors instructions from birth that orchestrate the timing and morphology of age-related structural changes. We hypothesized that by studying an informative inbred strain of mice, the aging DBA/2 strain, the molecular signatures of these age-related changes could be identified. Furthermore, these signatures could serve to construct a candidate genetic profile that may define those persons at risk for lung dysfunction with aging.

A limitation of previous surveys of organ-specific aging programs is the use of binary constructs of the aging phenotype, focusing on “young” versus “old”. Since the young organ is not necessarily the “control” for the old organ, we sought to develop an alternative approach to describe tissue aging. By performing a genome-wide transcriptional time course survey of the aging murine lung (over six time points), we were able to extract genes that not only displayed more complex patterns of expression with aging but also reflected known histologic events that could not be replicated by simple pair-wise comparisons. In this study, we focus on the gene cluster which corresponds to the transcriptional transition attending the onset of airspace enlargement, e.g. 8–12 months of age.

Previous genomic surveys of murine lung aging showed that 1) the terminal structural changes seen in the aged lung are associated with an altered transcriptome and 2) that the aging lung harbors both tissue-specific and aging specific molecular signatures. Misra and colleagues found that airspace enlargement in senescent DBA/2 mice is associated with the down-regulation of elastin and several collagen genes despite increased collagen content compared with the young adult controls [3], [4]. However, whether this pattern temporally approximated the onset of structural changes in the aging lung was not established. Thus, the senescent transcriptional program could reflect either an active pro-aging process or terminal changes in a failing tissue. Recently, Zahn reported tissue-specific transcriptomes, including the lung, of aging C57Bl/6 mice over four time points [5]. However, no correlation with architectural changes in tissues was pursued. These important findings augur a need for a more detailed assessment of the molecular signatures of aging lung pathology.

In this study, we show that airspace enlargement develops during the mid-range of the murine life-span and progresses through the late, preterminal time points and is accompanied by early oxidative stress, cell death and elastase activation. We also show that several genes are transiently induced during the onset of this structural change, and may possibly be the first signature of a tissue-specific aging program. This period, we further demonstrate, is punctuated by a marked, transient induction of immunoglobulin genes, accompanied by B lymphocyte (B-cell) activation/expansion, immunoglobulin deposition and macrophage infiltration. Taken together, our strategy has shown that time course data informed by structural surveys can reveal relevant pathways involved in tissue-specific aging that might be overlooked with conventional young-old pairwise analyses.

## Results

### Strategy of time course survey of the aging lung

**Figure 1:**
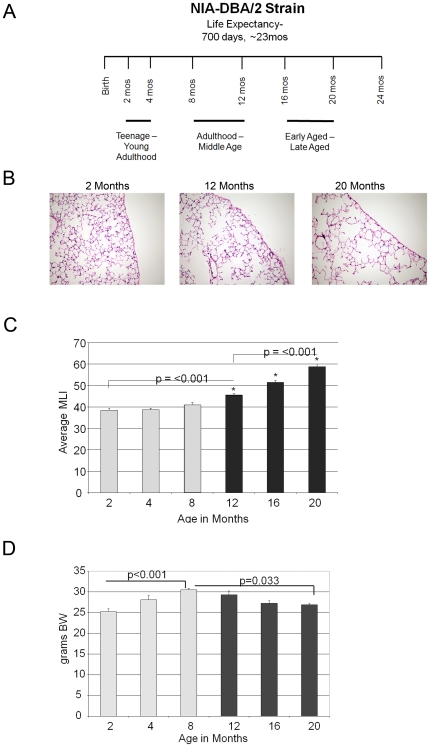
Time course analysis of aging lung phenotype. A. Schematic diagram of the lung harvest time points and the corresponding phases of human aging. B. Representative lung histology from hematoxylin and eosin stains of 2 month, 12 month and 20 month old mice depict a progressive increase in airspace size with age. Photomicrographs are representative of N = 4–6 mice per group. Original magnification 20×. C. Morphometric analysis of airspace caliber denotes transition point to progressive airspace enlargement between 8 and 12 months of age. N = 6 mice per group. D. Body weight measurement establishes trend to weight loss initiated between 8 and 12 months of age. N = 6 mice per group. MLI-Mean linear intercept (mm). BW-Body weight. The pale versus dark shades show different phases in the evolution of the noted parameter.

In order to delineate the critical signaling events which attend the onset of airspace enlargement during the aging of DBA/2 mice, we performed a detailed histologic and molecular analysis of the lungs of the mice at six different time points during adult life: 2 months, 4 months, 8 months, 12 months, 16 months and 20 months. These time points loosely correspond to specific maturation stages in humans: early adulthood (2–4 months), middle-age (8–12 months) and old age (16–20 months) (Figure 1A). At each time point, lungs were processed for histology, expression profile analysis and protein immunoblotting (Figure S1).

### Lung histology and morphometry in aging mice

We found a nonlinear pattern of airspace enlargement, denoted by MLI (mean linear intercept), that commenced at 12 months of age and progressed thereafter (Figure 1B,C). The airspace enlargement was homogeneous without any histologic stigmata of tissue destruction. Of note, the large vessels and microvasculature showed no evidence of morphologic change with aging. Consistent with the 8 to 12 month period representing a critical transition with respect to organismal aging, we found that the trend toward significant weight loss with age started at 12 months (Figure 1D). When MLI was adjusted for weight, a quadratic (but not linear) association was identified resulting in a p-value of 0.002 with an adjusted R^2^ of 0.28. Since weight has a bimodal curve morphology with age, we considered whether analysis of mice ≥8 months of age might show an independent association between MLI and weight. The observed linear association between MLI and weight in this group, however, was eliminated with adjustment for age (p = 0.68). A significant association existed between MLI and age (R^2^ 0.89) with no evidence of improved association when weight was included in the model. Taken together, these data show that even when corrected for weight, age remains an independent factor contributing to MLI.

### Measures of lung injury with aging

**Figure 2:**
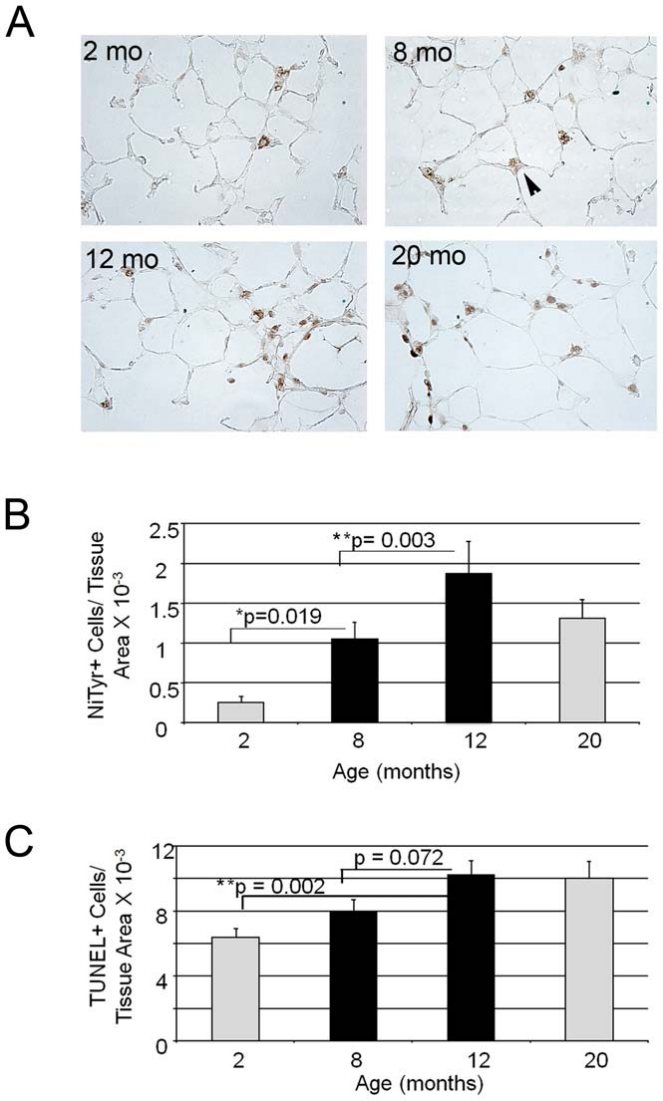
Increased oxidative stress and cell death evident in murine mid-life. A. Representative immunohistochemical staining for nitrotyrosine in mice at 2 months, 8 months, 12 months and 20 months of age. Robust staining is evident by 8 months of age. Site of staining is in alveolar epithelial cells (arrowheads). N = 4–6 mice per group. Original magnification, 20×. B. Quantitative immunohistochemistry of nitrotyrosine staining shows enhanced staining in the 8 month old lung which progresses through later time points. N = 6 mice per group. C. Quantitative immunohistochemistry of TUNEL staining of lungs from aging mice shows increased cell death at 8 months of age which persists through later time points. N = 6 mice per group. Reported data are mean values +/− SEM from at least five mice for each group. *p<0.05, **p<0.01.

Airspace enlargement frequently accompanies various forms of lung injury that result in oxidative stress, cell death, reduced proliferation and/or local inflammation. Increased oxidative stress is a signature of systemic aging and likely contributes to the higher incidence of malignancy, fibrosis and low-grade inflammation in elderly persons [6], [7]. Immunohistochemical staining for nitrotyrosine, a marker of oxidative stress, revealed a progressive increase in oxidative stress from 2 month to 20 months of age (Figure 2A,B). The site of the staining was in the airspace epithelial compartment, especially type II cells. Because iNOS (inducible Nitric Oxide Synthase) activity is frequently associated with inflammation-associated oxidative stress, we examined iNOS expression by immunoblotting in the aging lung lysates. We saw no increase in iNOS expression in the aging lung (data not shown). We also measured cell death in the airspace compartment by performing TUNEL (Terminal Transferase dUTP Nick End Labeling) staining. We saw a different temporal pattern of staining, compared with nitrotyrosine, with a statistically significant enhancement in staining evident between 2 months and 12 months (Figure 2C). Using a caspase 3 bioassay, we saw no evidence of increased caspase activity in the aging lungs until 20 months of age (Figure S2). This suggests that the early cell death represents either caspase-independent apoptosis and/or necrosis. Thus oxidative stress appears to precede both the development of exuberant cell death and significant airspace enlargement in the aging lung. Of note, the levels of oxidative stress in the aging lung is much less than that observed in other models of airspace disease such as the tight-skin mouse [8].

### K-Means Clustering Profiles

**Figure 3:**
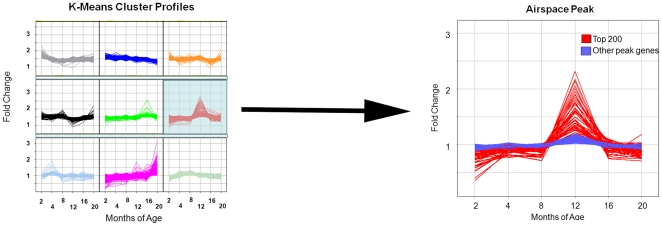
Schematic of selection of K-means cluster profile that temporally approximates airspace enlargement. Nine profiles generated by random selection for difference from evenly spaced profiles are depicted. The profile whose peak corresponded to the onset of airspace enlargement is enlarged on the left. Red shows the top 200 genes. Blue shows the remainder of genes within the cluster.

Nine clustering profiles of the time course transcriptome were generated using a K-means strategy. Of the nine cluster patterns, one was selected for further interrogation based on the timing of induction consistent with the onset of the airspace phenotype (Figure 3A, B). This cluster contained 2220 genes out of the 25,000 known genes on the Illumina chip. The genes in this cluster were termed the “airspace peak”. The top pathways identified by Gene Ontology included those involved in humoral immune response, transport, negative regulation of cell cycle and response to wounding (Table 1). The top 200 genes in the cluster are shown in Table S1. Confirming the pathway analysis, immunoglobulin genes were disproportionately represented in this peak (25 of top 50 genes). We selected eight genes represented in the top 100 within the airspace peak for further validation in triplicate by real time RT-PCR (reverse transcriptase polymerase chain reaction). Since our gene list is based on a profile over six time points, we recognize that two-point comparison is not a true validation. We elected to use three of these comparisons for these eight genes spanning the 4 month to 12 month interval, 4 vs 8, 4 vs 12 and 8 vs 12. We specifically chose non-immunoglobulin genes for this validation given the known overlapping specificity of immunoglobulin transcript analysis. Five out of the 8 genes surveyed in the airspace peak were increased by RT-PCR (Table S2).

**Table 1:** Overrepresented pathways within airspace peak.

Pathway Representation (GO)	p-value
Humoral Immune Response	9×10^−5^
Transport	5×10^−5^
Negative regulation of cell cycle	1×10^−4^
Response to wounding	1×10^−4^

### Excessive immunoglobulin synthesis and deposition in the aging lung

**Figure 4:**
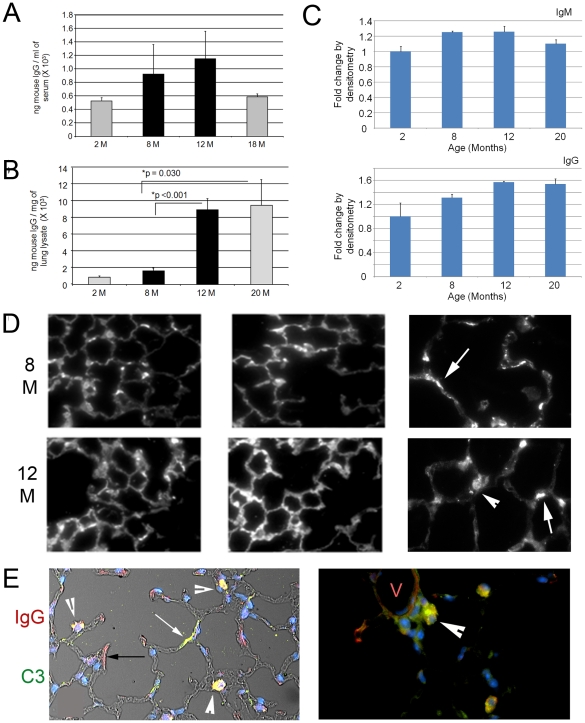
Immunoglobulin deposition in lungs of aging mice. A. ELISA analysis of IgG containing complexes in serum from mice at designated ages. A trend towards a significant increase is apparent between 8 and 12 months of age. N = 6 mice per group. B. ELISA analysis of lung lysates from mice at different ages. Data are mean +/− SEM. A significant increase in immunoglobulin deposition in the lung occurs between 8 and 12 months of age. N = 4–6 mice per time point. C. Densitometric analysis of immunoglobulin expression in lung lysates from mice at the denoted ages show that IgM (top panel) and IgG (bottom panel) are induced in the 8 and 12 month old lung, respectively. D. Immunohistochemical staining for IgG in lungs of representative mice at designated ages. Arrows denote immunoglobulin deposition in airspace wall. Arrowheads denote cell-associated deposition. N = 6–8 mice per group. A generalized increase in deposition in the airspace compartment occurs in the 12 month old lung. All images are 20×, except for insets on right, which are 40×. E. Coimmunofluorescent staining for IgG (red) and C3 complement (green) with phase overlay (L) shows evidence of granular colocalization (white arrowheads) in alveolar epithelial cells along with sites of predominant IgG deposition (black arrow) and C3 deposition (white arrow). Coimmunofluroescent staining for IgG and C3 complement in 12 month old lung shows strong colocalization in the perivascular region. V-vessel lumen. 40× magnification.

Although immunosenescence involves overall dampened immune responses with aging, autoimmunity is an observed accompanying pathology [9]. In fact, this low grade inflammation has been termed inflama-aging and may contribute to organ-specific disease pathology [10], [11]. We explored whether the exuberant immunoglobulin production observed in the 12 month old lung suggested that immunoglobulin deposition might contribute to the airspace pathology. ELISA (enzyme-linked immunosorbent assay) analysis of serum showed that a trend towards a significant increase in IgG (immunoglobulin G) in serum was present in the 8 and 12 month old specimens compared with the 2 or 18 month old samples (Figure 4A). Analysis of lung tissues, by contrast, demonstrated a significant increase in immunoglobulin deposition in the 8 and 12 month old samples (Figure 4B). Quantitative densitometry of immunoblots of whole lung lysates for IgG and IgM also showed an induction of 25 and 55 kDa bands at the 8 and 12 month time points (Fig. 4C and Figure S3). Immunohistochemical staining for mouse IgG showed that in 8 month old mice, compared with 2 month old mice, there was increased staining for these complexes in the septal walls (Figure 4D top panel and data not shown). By 12 months of age, a marked increase in staining was evident in both epithelial cells and in the septal walls (Figure 4D bottom panel). Colocalization of IgG and complement C3 confirms immune complex deposition in the 12 and 20 month old lungs that is predominantly cell-associated (Figure 4E). Thus, immunohistochemistry, ELISA, immunoblotting and transcriptional analysis all support an elaboration of immunoglobulin production and deposition in the presenescent lung parenchyma at a time point that coincides with structural changes in the airspace morphology.

### Immune cell dysregulation accompanies lung simplification

**Figure 5:**
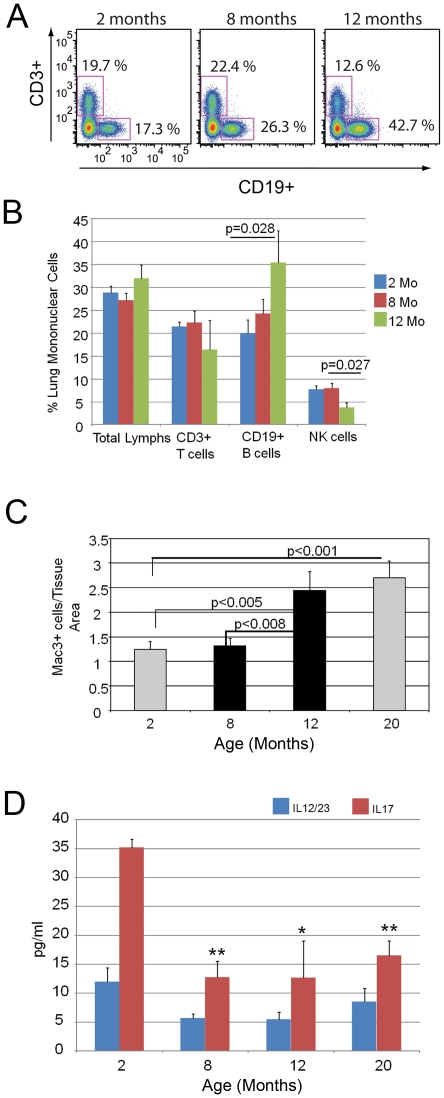
Alterations in immune cell compartments in the aging lung. A. Representative histograms depicting lymphocyte subsets identified in lungs of mice at indicated ages. An increase in CD19+ cells occurs at 12 months of age compared with earlier time points (2 and 8 months). N = 4–6 mice per time point. B. Relative proportion of lymphocyte subsets quantified by flow cytometry in lung mononuclear cells isolated from mice at designated ages. N = 4–6 mice per time point. C. Quantitative immunohistochemistry of macrophage abundance in lungs of aging mice. An increase in macrophage infiltration occurs at 12 months of age compared with 8 months. Data are mean +/− SEM. N = 3–6 mice per time point. D. IL12/23 and IL17 ELISA analyses of lung lysates from mice at designated ages. A reduction of IL17 levels is evident in the lungs of mice 8 months of age and older compared with 2 month old mice.

Flow cytometry on whole lung preparations and BAL (bronchoalveolar lavage) specimens from 2 months, 8 months, 12 month and 20 month old lungs showed an increase in B-cell content between 8 and 12 months (Figure 5A,B). B-cell immunophenotyping showed an increase in CD86+ and TLR4+ (toll receptor 4+) lymphocytes attending the 8 to 12 month transition (Table 2). Since CD86 is a costimulatory molecule that is induced in activated lymphocytes, these data are consistent with B-cell activation accompanying the airspace simplification observed in the aging lung. No change in the abundance of total T-cells, CD4+, or FoxP3+ T regulatory subsets was seen (Figure 5A,B and Figure S4B). Interestingly, a modest increase in the CD8+ compartment was evident between 2 months and 8 months and maintained at 12 months (Figure S4A). Despite the lack of a triggering insult, we examined macrophage influx and activation in the aging lungs. We found that macrophages were increased in the 12 month lung compared with the 8 month time point (Figure 5C). Flow cytometric evaluation showed an increase in MHCII (major histocompatibility complex class II) expression in the monocyte compartment at 12 months of age, reflecting an activated phenotype (Table 2).

**Table 2:** Flow cytometric analysis of mononculear cell subsets in aging lungs.

*% B Lymphocytes*	8 month	12 month	p-value
**CD86+**	12.4	16.0	0.017*
**MHC-II+**	80.4	83.5	0.322
**TLR2+**	18.8	28.7	0.065
**TLR4+**	18.1	33.5	0.010*

*% Monocytes*	8 month	12 month	p-value
**CD11b/c+**	3.68	5.79	0.118
**CD40+**	31.2	43.5	0.195
**CD86+**	28.9	29.7	0.870
**MHC-II+**	33.7	49.4	0.048*
**TLR2+**	17.0	19.1	0.629
**TLR4+**	14.8	28.2	0.168

*p<0.05.

Autoimmunity has been proposed as a component of immunosenescence, however to date no culprit immunogenic lung proteins have been identified and the lung pathology accompanying most rheumatologic illnesses is fibrosis rather than airspace enlargement. We examined whether selected autoimmune cytokines (IL17, IL12/IL23heterodimer) were induced in the aging lung [12], [13]. We saw a reduction in lung levels of these cytokines from 8 months to 20 months when compared to 2 month old samples (Figure 5D). Although these results do not eliminate an autoimmune contribution to the immunoglobulin deposition and airspace enlargement, they suggest that the more conventional cytokines participating in autoimmunity are downregulated.

Taken together, these data show that an induction of B-cell expansion/activation and immunoglobulin production in the aging lung accompanies the earliest stage of airspace enlargement. Further, the immunoglobulin deposition is associated with an influx of macrophages.

### Matrix deposition and turnover in the aging lung

**Figure 6:**
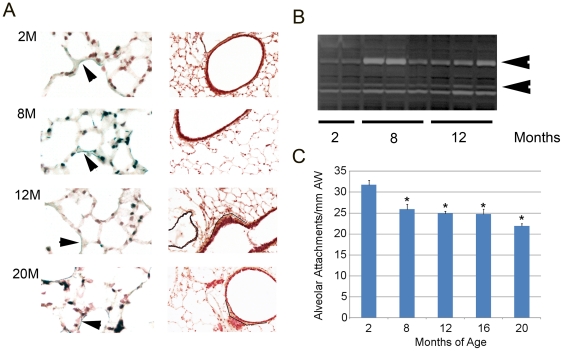
Matrix localization in aging lung. A. Movat's staining of lungs from mice at designated ages. Left panel shows representative high power (40×) images of lung parenchyma. Right panel shows low power (10×) images of bronchioles. Arrowheads denote sites of elastin deposition (blue-black). Line demarcation indicates areas of enhanced collagen content (yellow). Elastin deposition in the alveolar walls is modestly reduced at 12 months but shows recovered abundance at 20 months. Peribronchiolar collagen deposition is increased in the 12 month and 20 month old lungs. N = 4–6 mice per time point. B. Zymographic analysis of lungs from mice at the designated ages. Top arrow denotes MMP9 band. Bottom arrow denotes MMP2 band. N = 4–6 mice per time point. C. Quantitation of alveolar attachments in the lungs of mice at the designated ages show a progressive reduction in attachments with increasing age. N = 4–6 mice per time point. *p<0.05 compared with 2 months.

**Figure 7:**
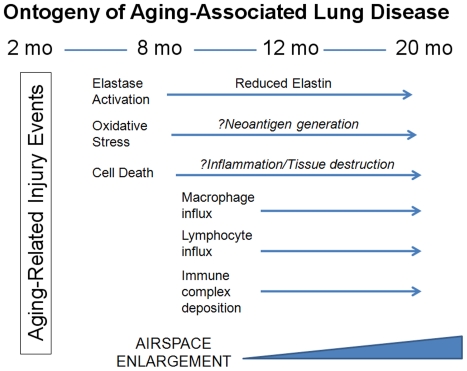
Schematic depiction of the ontogeny of injury events associated with airspace enlargement in the aging murine lung.

Our finding of early oxidative stress followed by immune cell infiltration and immunoglobulin deposition prompted us to determine whether the enhanced oxidative stress might contribute to matrix turnover that could trigger an immune response. Recent reports show that both aging and non-aging related redox changes can alter matrix homeostasis [14], [15]. We first assessed elastin and collagen content and localization in the aging lung by Movat staining. Both matrix elements were preserved in localization and morphology across the aging time frame of 2 months to 20 months (Figure 6A). Of note, we did see reduced elastin deposition in the airspace wall at 12 months when compared to 8 months of age. Increased peribronchiolar collagen deposition in the 12 month and 20 month old mice was consistent with previous observations of the aging rodent lung [3]. Although we saw no overt evidence of elastin fragmentation or discontinuity, usual features of elastase-associated tissue destruction, in the 12 or 20 month lungs, we performed zymography to assess lung elastase activity. Zymography showed an early increase in MMP9 activation in the 8month old lung that was maintained at 12 months (Figure 6B). By contrast, MMP12 expression decreased with age, suggesting that MMP12, a known contributor to cigarette smoke induced emphysema in murine models, is not the source of elastin turnover with murine aging (Figure S5). A progressive reduction in the number of airway alveolar attachments, a signature of alveolar wall destruction, was observed from 8–20 months of age (Figure 6C). These data suggest that early elastase activity is not only destructive and precedes the onset of airspace enlargement, but is also a plausible trigger for the immunoglobulin liberation and macrophage influx that initiates the airspace lesion (schematicized in Figure 7).

## Discussion

Airspace enlargement is a well-recognized pathological signature of respiratory aging [3], [16]. Whether this process recapitulates or incorporates known pathways of organismal aging is unknown. We sought to delineate the molecular profile of age-related airspace enlargement by performing a time course transcriptional survey of the aging DBA/2J lung throughout adult life. We found a distinct gene induction pattern punctuated by immunoglobulin production and cell cycle dysregulation which attended the onset of airspace enlargement. Whereas cell cycle changes have been linked to organismal aging, we show a novel role of oxidant-triggered matrix remodeling and dysregulated lymphocyte function in the aging lung.

Despite the fact that airspace enlargement is a known feature of the aged lung, whether the lesion develops from injury or simply reflects reduced matrix abundance in tissue is a subject of debate. Recent observations, including those reported here, support the former paradigm. The oxidant injury, elastase activation and cell death preceding the onset of airspace enlargement in our studies strongly implicate age-associated tissue stressors. Consistent with this paradigm, Sato reported that SMP30-deficient mice, a proposed model for the “senile lung”, not only develop accelerated age-associated airspace enlargement but also display increased oxidative stress, cell death and susceptibility to cigarette smoke induced pulmonary emphysema [17].

Several investigators have shown or postulated reduced immune function in aged persons and in murine models of aging [18], [19], [20]. Alterations in immune responses with aging likely contribute to the increased susceptibility to infectious insults and malignancy in elderly persons. Unfortunately, no unifying pattern of changes has been reported in humans or rodent models. Defects in humoral immunity can accompany aging, manifest in both reduced specific antibody responses and enhanced nonspecific antibody production [21], [22]. Impaired function of hematopoietic stem cells in the aging bone marrow seems to result in both reduced production of naïve B-cells and marked restriction of the B-cell immune repertoire in a murine system [23]. Elevated serum immunoglobulin levels and increased antibody-producing cells in the spleen and bone marrow have also been reported in aging mice [24]. We found that the upregulation of immunoglobulin genes during aging-related airspace enlargement is accompanied by B-cell expansion, B-cell activation and enhanced synthesis and deposition of immunoglobulin complexes in the lungs of aging mice. B-cell activation is a feature of several chronic inflammatory conditions such as COPD (chronic obstructive pulmonary disease), rheumatoid arthritis and multiple sclerosis. An increased number of lymphoid follicles in the airway submucosa has been identified not only in mice exposed to cigarette smoke, but also in patients with advanced emphysema [25], [26]. Others have invoked a pathogenic contribution of parenchymal and airway lymphoid collections, possibly representing an exaggerated immune response triggered by microbial, matrix or tobacco smoke antigens [26]. A recent study showed an increase in autoantibodies directed against both lung epithelial and endothelial determinants in the serum of patients with COPD [27]. In our aging lung model, there is no exposure to known airspace insults like microbes or tobacco smoke; nonetheless, oxidative stress, elastase activation and epithelial cell death occur. Thus, the trigger for the elaboration of immunoglobulins in the aging lung may involve oxidative stress promoted matrix degradation, an established mechanism for aging-associated tissue remodeling [14], [15]. While no significant matrix turnover is evident by histochemical staining at the 12 month time point, our zymography data suggests that low-level turnover is present at 8 months and may be sufficient to drive the initiation of the immune response (Figure 6B).

We propose three possible mechanisms connecting the immune signature with the airspace lesion (summarized in Figure 7). First, the enhanced elastase activity in the absence of histologic evidence of tissue damage might generate matrix degradation products that are immunogenic. This is a proposed but imperfectly supported mechanism for emphysema [27]. The critical omission in the theory is the identification of a consistently triggering matrix-derived antigen. A second possibility is that an alteration in immunosurveillance at midlife could create a permissive environment for an immune response to develop to a variety of stimuli. Since the lung is an organ that is constantly exposed to foreign antigens, any impairment in immune function can translate into dysregulated innate and adaptive responses to antigen and lung-specific pathology. However, immunomodulatory mechanisms may be sufficiently preserved that the dysregulated response is eventually arrested. By this view, the ongoing airspace enlargement, oxidative stress and cell death manifest a tissue-specific inability to repair/regenerate the airspace compartment and low grade inflammation as reflected by macrophage infiltration. This paradigm is quite similar to the progressive airspace and airway pathology observed after smoking cessation in persons with COPD/emphysema. A third possible mechanism is that a primary alteration in oxidant/antioxidant balance, conferred by midlife, results in the generation of neoantigens secondary to oxidation of resident proteins in the lung, an organ exquisitely susceptible to oxidant injury. Such neoantigens could trigger a local immune response and initiate the sequelae described above. Both of these mechanisms rely on a relatively preserved immunomodulatory axis. Consequently, an active direction of our lab is the dissection of these immunomodulatory pathways as they relate to lung aging.

The studies presented here demonstrate two intriguing findings. First, we show that aging associated airspace enlargement develops during middle age and that a contemporaneous innate and adaptive immune signature heralds its onset. This signature consists of exuberant immunoglobulin production, B-cell activation, local immunoglobulin deposition and macrophage infiltration. Second, we demonstrate that early aging-associated oxidative stress and elastase activation precedes overt inflammation, immunoglobulin deposition and airspace enlargement. We present a novel pathogenetic scheme for aging-associated airspace enlargement with presenescent oxidative stress triggering both canonical and noncanonical mechanisms of emphysema. These findings suggest that the crucial point of intervention for aging related lung dysfunction may be well before airspace disease is clinically apparent.

## Materials and Methods

### Animal Work

Aged male DBA/2 mice (2–20 months of age) were obtained from the specific pathogen-free Charles River-National Institute of Aging (NIA) facility. These mice were temporarily housed in a Johns Hopkins Medical Institution mouse facility accredited by the American Association of Laboratory Animal Care until time of euthanization. The animal studies were reviewed and approved by the institutional animal care and use committee of Johns Hopkins School of Medicine.

### RNA Extraction and Illumina Chip Hybridization

Total RNA was extracted from murine lung using the Trizol Reagent method (Invitrogen, Carlsbad, California 92008, cat. no. 15596-026). The six RNA samples from each time point were pooled into two groups comprised of three murine specimens. RNA samples were labeled and hybridized to Illumina Sentrix MouseRef-8 Expression Beadchips (Illumina, San Diego, CA 92121-1975, cat.no. BD-26-201) according to manufacturer's protocol.

### Data normalization

The microarray data was normalized with *Affy*, a Bioconductor package (http://www.bioconductor.org), utilizing the quantile normalization method to reduce the variation between microarrays that can develop during the processes of sample preparation, manufacture, fluorescence labeling and hybridization [28]. Assuming that there is an underlying common distribution of signal intensities across microarrays, the quantile normalization method makes the distribution of signal intensities for each microarray in a set of microarrays the same by forcing the values of quantiles to be equal. An underlying assumption of the quantile normalization method is that only a small fraction of genes is differentially expressed between the sample conditions. When analyzing the gene expression changes with age within individual tissue types, normalization was separately made for the data of each tissue type in order to avoid any “reduced” differences that could be introduced by normalizing the data across tissue types together. With the normalized signal data, principal component analysis (PCA) was performed in *R* to assess sample variability.

### K-Means Clustering

Differentially-expressed genes were categorized using modified Best K-Means clustering (Spotfire 9.1.1). The number of clusters was chosen empirically based on visual inspection as differing from evenly spaced profiles. To reduce the background and ensure the clustering quality, only genes with detectable hybridization signals in the arrays from all aging groups were included. Nine profiles were generated using Spotfire cluster initialization with a data centroid based sea. One cluster was selected for further analysis based upon a peak transcriptional induction coincident with the onset of airspace enlargement. Genes within this cluster were ranked based on similarity to exemplar and examined by Gene Ontology Definition for pathway assignment. The top 200 genes, ranked by similarity to exemplar, are shown in Table S1.

### Functional Classification

Differentially expressed genes were classified into functional categories based on the Gene Ontology (GO) definition using publicly available web-based tools Onto-express and David (data base of annotation, visualization, and integrated discovery). For each level of annotation, the calculated p-value represents the probability that the specific gene-function was randomly distributed between groups [29].

### Real Time PCR

Total RNA isolated from lung tissues was treated with DNase and reverse-transcribed using a first-strand DNA sysnthesis kit from Invitrogen. The PCR was performed on an ABI Fast 7500 System (Applied Biosystems, Foster City, CA). TaqMan probes for the respective genes were custom-generated by Applied Biosystems based on the sequences in the Illumina array and used per manufacturer's instructions. The expression levels of target genes were determined in triplicate from the standard curve and normalized to Gapdh mRNA level.

### Western Blot Analysis

Western blot analysis was performed using standard methods. Primary antibodies and dilutions were as follows: IgG1 (goat polyclonal, Abcam, 1∶1000), IgA (goat polyclonal, Abcam 1∶1000), IgM (goat polyclonal, Abcam 1∶1000), CTGF (Abcam 1∶5000), psmad2 (Cell Signaling 1∶1000), iNOS (Abcam 1∶200), beta-actin (rabbit polyclonal, Abcam, 1∶1000), MMP12 (Santa Cruz, 1∶250).

### Histology, Morphometry and Immunohistochemistry

Histologic, morphometric and immunohistochemical methods were as described previously [8]. Alveolar attachments were quantified in a blinded fashion from 20× H&E images and normalized to airway perimeters. Antibodies were used at the following concentrations: Nitrotyrosine (mouse monoclonal, Abcam, 1∶250), MAC-3 (rat monoclonal, BD Pharmingen, 1∶100), Neutrophil (rat monoclonal, Serotec 1∶50), psmad2 (rabbit polyclonal 1∶5000) and SP-D (mouse monoclonal, Santa Cruz, 1∶200). Movat's stain was performed on selected sections utilizing a standard protocol.

### Apoptosis Assays

TUNEL staining was performed using the Calbiochem TdT-FragEL DNA Fragmentation Detection Kit per standard protocol as published [8]. Caspase 3 activity was measured in whole lung lysates using the Promega Caspase Glo 3/7 Assay kit.

### ELISA assays

Serum samples and lung lysates from mice at designated ages were subjected to Mouse IgG ELISA analysis per Roche protocol. IL17 and IL12/23 ELISA assays were performed per Invitrogen and R&D protocols, respectively.

### Isolation of Lung mononuclear cells

Lungs were minced and incubated at 37°C and single cell suspensions were prepared. Lymphocytes were gated with characteristic low forward scatter/side scatter, using a FACSAria instrument and FACSDiva for data acquisition (Becton Dickinson), and Flowjo for analysis (Tree Star Inc) as previously published [30].

### Zymography

Lung tissue lysates were prepared in a cold room at 4C. Tissue was homogenized in 50 µL PBS and centrifuged at 14000 RPM for 20 min. The supernatant was removed and used as sample lysates. Fifty µg of lung lysates were loaded on a10% Criterion Zymography Precast Gel (Biorad) and run at 120 V. Twenty-five µL of recombinant mouse MMP9 protein (R&D Systems, Minneapolis, MN) was loaded as a positive control. The gel was soaked in 1× Renaturing Buffer (Biorad) twice for 30 minutes each at room temperature and incubated in 1× Development Buffer (Biorad) overnight at 37C. The gels were stained with Coomassie Brilliant Blue R-250 Staining Solution (Biorad), followed by 1× Destain Coomassie R-250 Solution (Biorad) until a clear band appeared against a blue background.

### Data Analysis

Results are expressed as means ± SEM unless otherwise stated. Screening comparisons across multiple time points were performed by one-way ANOVA. These were followed by pairwise-comparisons using the two-sample t-tests or Mann-Whitney rank sum tests. All statistical analyses were performed with Sigmastat (version 3.5; Systat Software, Chicago, IL). A p<0.05 was considered significant.

## Supporting Information

Figure S1 **Strategy for analysis of lung phenotype at different ages.**
(TIF)

Figure S2 **Caspase activity in the aging lung.** A. Caspase activity in lung lysates from mice at designated ages. N = 4–6 mice per time point. *p<0.01.
(TIF)

Figure S3 **Immunoglobulin expression in the aging lung.** Western blotting for immunoglobulins in lung lysates from mice at designated ages. Top-IgM blot, Bottom-IgG blot.
(TIF)

Figure S4 **T cell subsets in the aging lung.** A. Relative Proportion of CD4+ and CD8+ cells in CD3+ lymphocyte subset quantified by flow cytometry in lung mononuclear cells isolated from mice at designated ages. N = 4–6 mice per time point. B. Relative proportion of FoxP3+ cells in CD4+ lymphocyte subset from mice at designated ages. N = 4–6 mice per time point. Asterisk designates p<0.05 compared with 2 month time point.
(TIF)

Figure S5 **MMP12 expression in the aging lung.** Densitometric analysis of MMP12 protein expression in lung lysates from mice at designated ages normalized to actin. N = 4–6 mice.
(TIF)

Table S1 **Top 200 genes in airspace peak.**
(PDF)

Table S2 **Real-time PCR validation of genes in airspace peak.**
(PDF)
