# Supplementary figures and images for: Critical Transition in Tissue Homeostasis Accompanies Murine Lung Senescence
Source: PLoS One. 2011 Jun 21;6(6):e20712. doi: 10.1371/journal.pone.0020712 (PMC3119663; doi:10.1371/journal.pone.0020712)

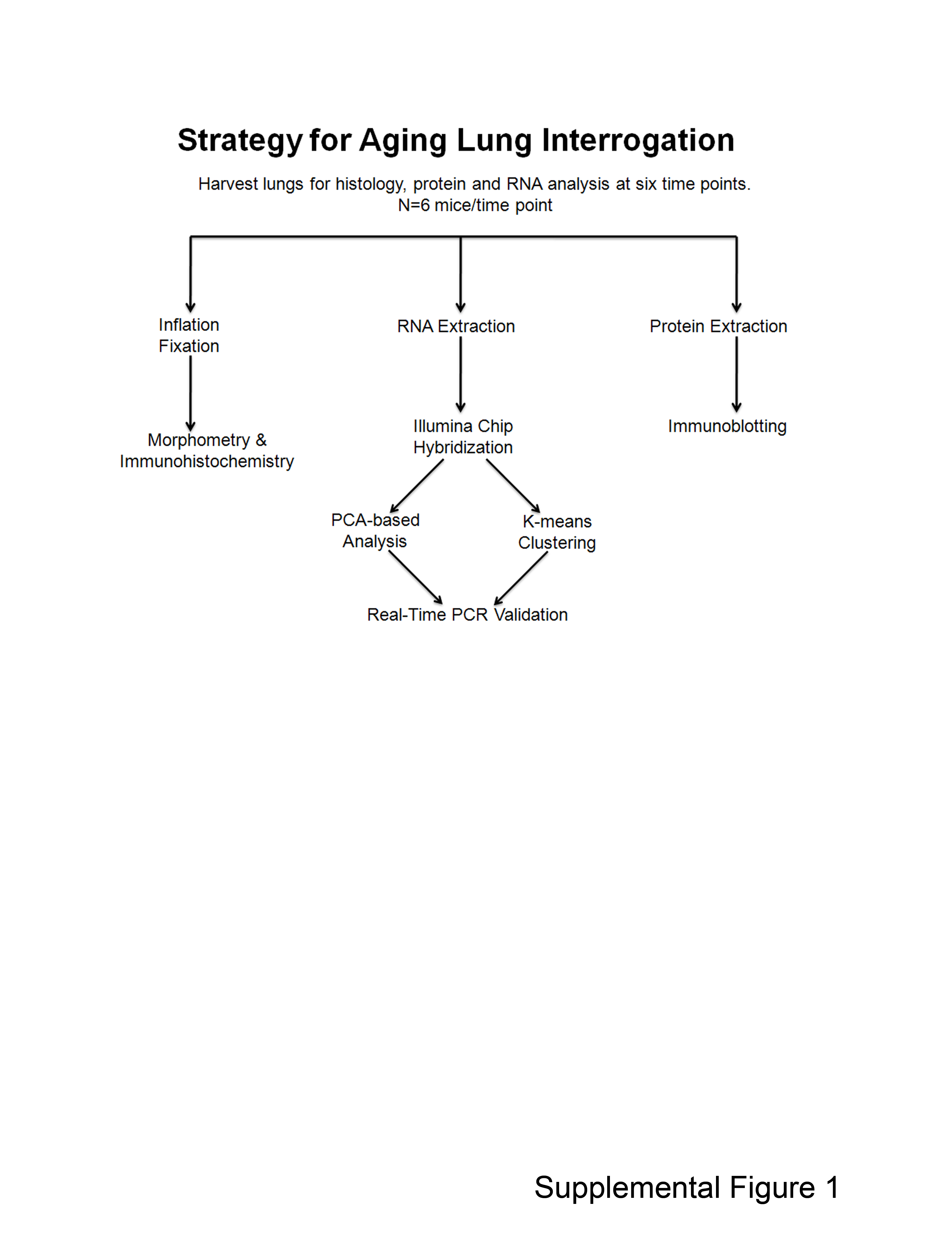

Supplement: Figure S1 — Strategy for analysis of lung phenotype at different ages. (TIF) [file pone.0020712.s001.tif]

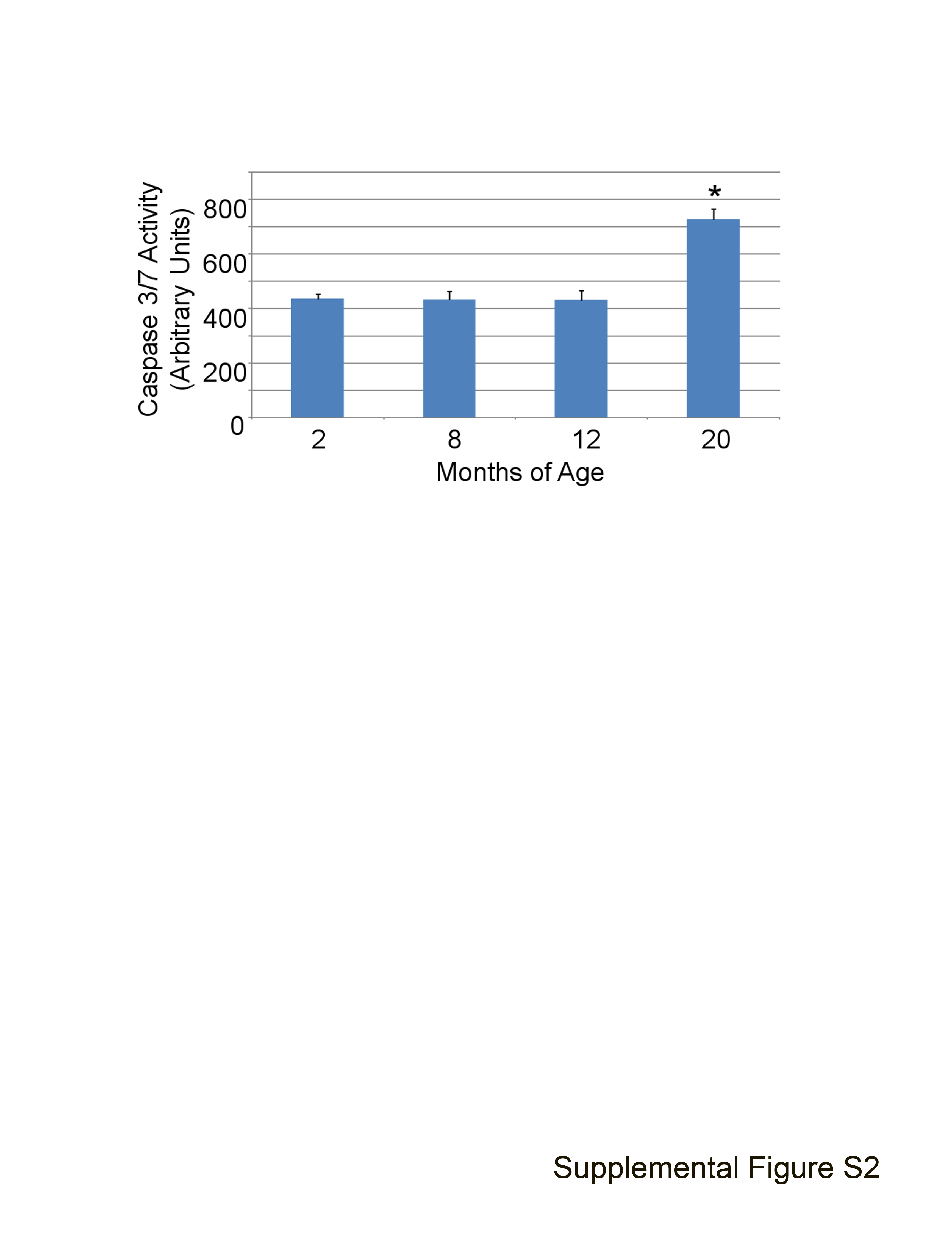

Supplement: Figure S2 — Caspase activity in the aging lung. A. Caspase activity in lung lysates from mice at designated ages. N = 4–6 mice per time point. *p<0.01. (TIF) [file pone.0020712.s002.tif]

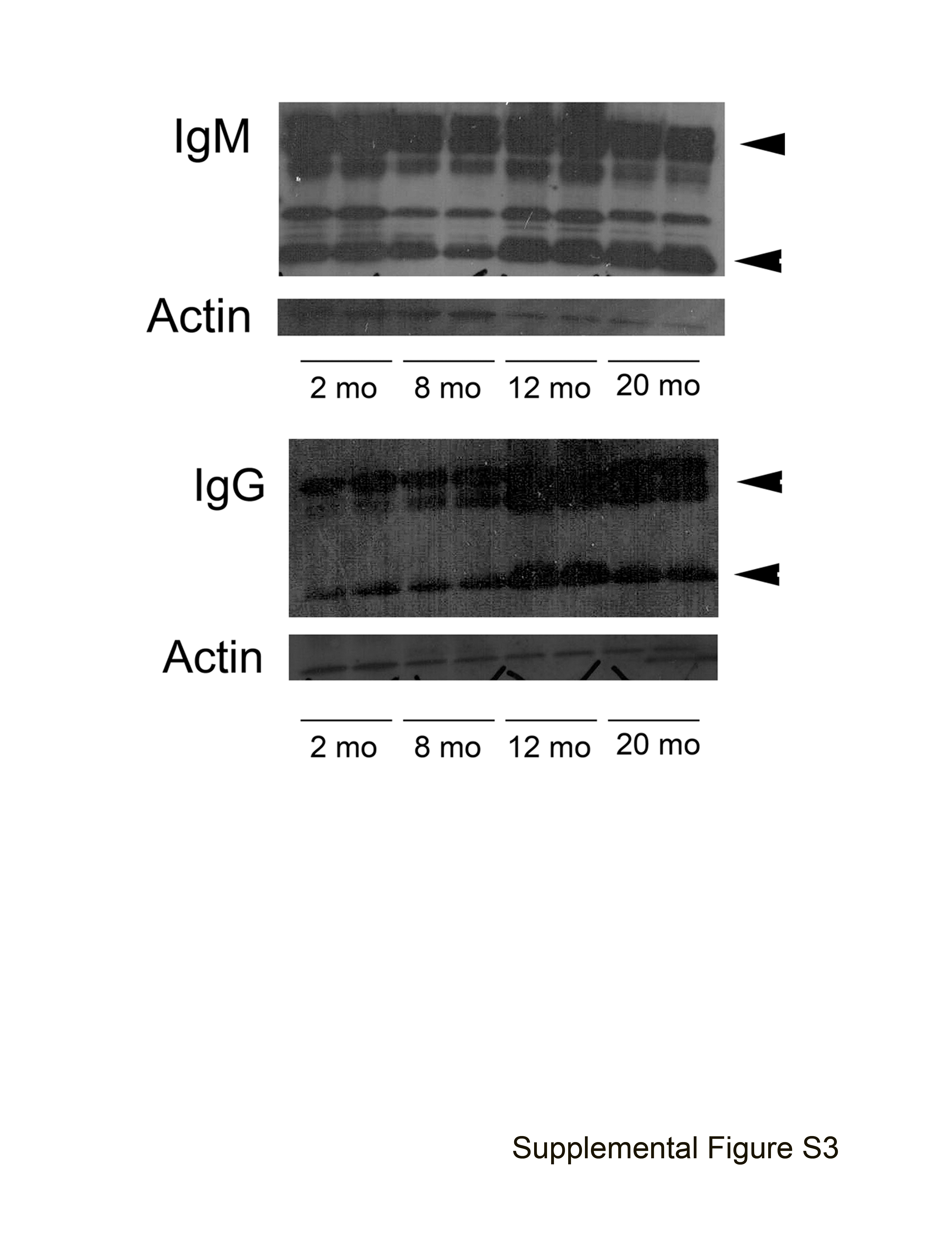

Supplement: Figure S3 — Immunoglobulin expression in the aging lung. Western blotting for immunoglobulins in lung lysates from mice at designated ages. Top-IgM blot, Bottom-IgG blot. (TIF) [file pone.0020712.s003.tif]

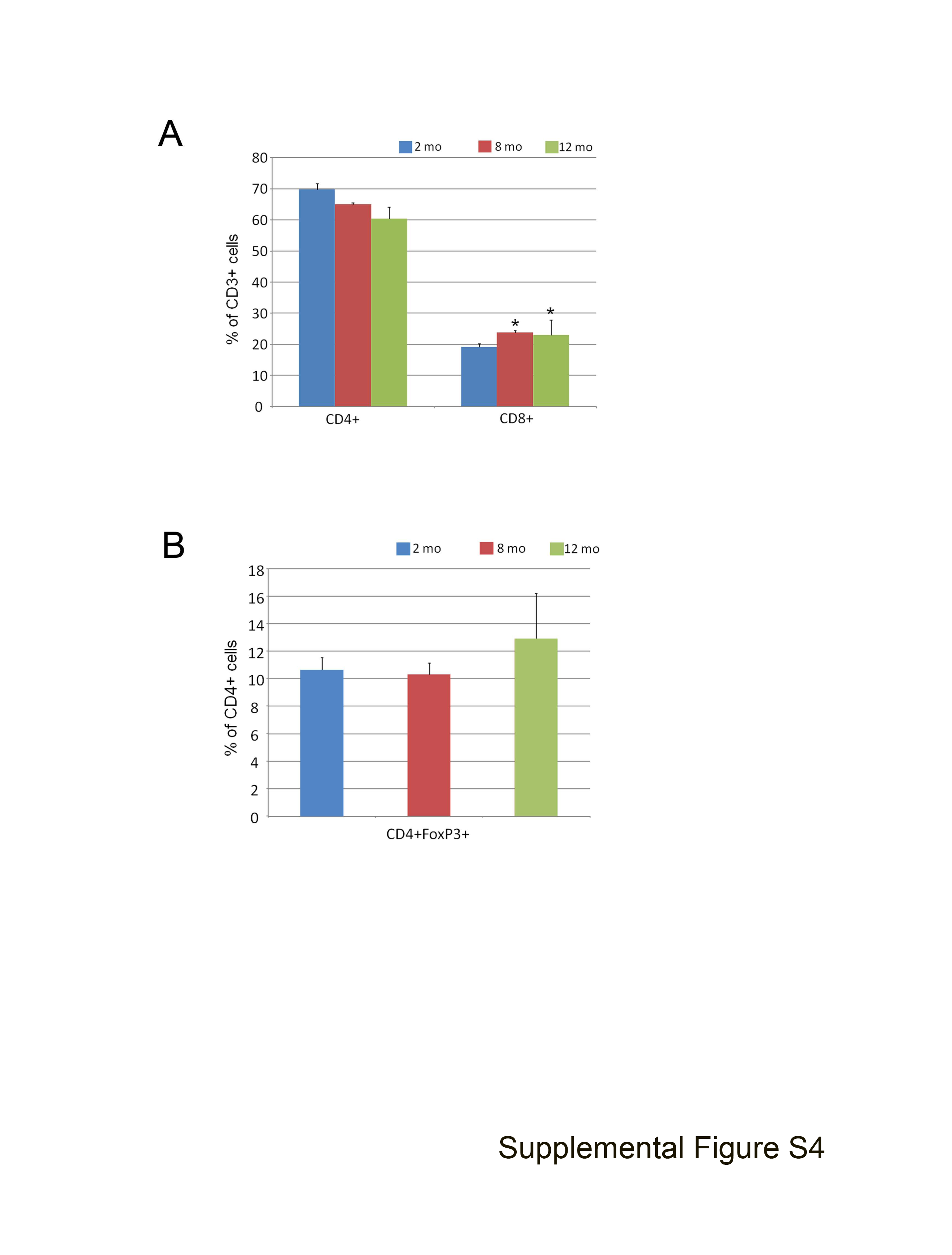

Supplement: Figure S4 — T cell subsets in the aging lung. A. Relative Proportion of CD4+ and CD8+ cells in CD3+ lymphocyte subset quantified by flow cytometry in lung mononuclear cells isolated from mice at designated ages. N = 4–6 mice per time point. B. Relative proportion of FoxP3+ cells in CD4+ lymphocyte subset from mice at designated ages. N = 4–6 mice per time point. Asterisk designates p<0.05 compared with 2 month time point. (TIF) [file pone.0020712.s004.tif]

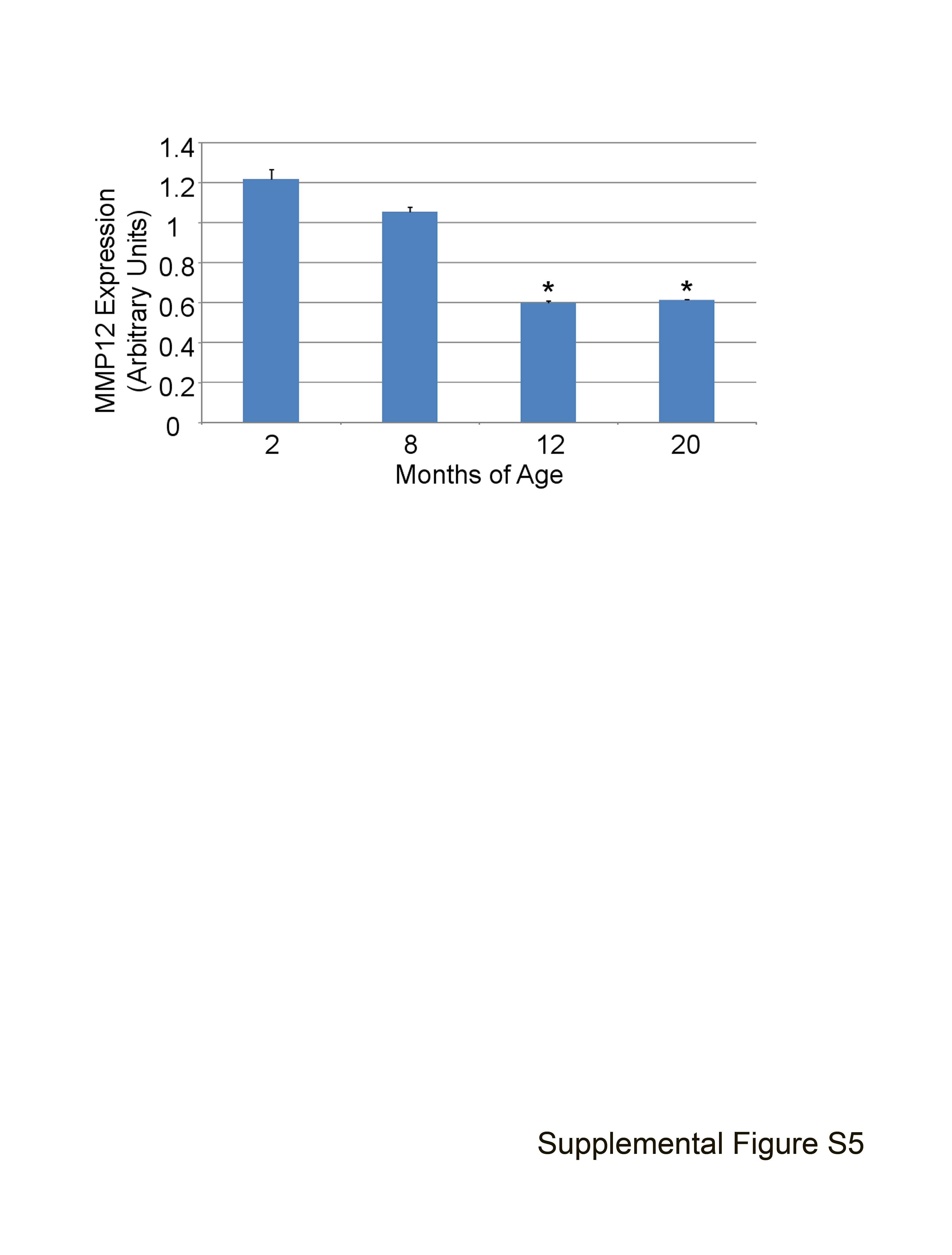

Supplement: Figure S5 — MMP12 expression in the aging lung. Densitometric analysis of MMP12 protein expression in lung lysates from mice at designated ages normalized to actin. N = 4–6 mice. (TIF) [file pone.0020712.s005.tif]
