# Supplementary material for: Critical Transition in Tissue Homeostasis Accompanies Murine Lung Senescence
Source: PLoS One. 2011 Jun 21;6(6):e20712. doi: 10.1371/journal.pone.0020712 (PMC3119663; doi:10.1371/journal.pone.0020712)
Supplement: Table S1 — Top 200 genes in airspace peak. (PDF) [file pone.0020712.s006.pdf]

Supplemental Table 1: Top 200 Genes in Airspace Peak

Probe Search_key	Rank	Probe _Id	Entrez _ID	Gene Symbol	Gene Name
IGKV6-17_Y15978_Ig_kappa_variable_6-17_13	1	ILMN_2674666	676136	LOC676136	similar to Ig kappa chain V-V region MPC11 precursor (LOC676136), mRNA.
IGHV1S13_X00160$K00706_Ig_heavy_variable_1S13_8	2	ILMN_1259003	637785	LOC637785	similar to Ig heavy chain V region 3 precursor (LOC637785), mRNA.
IGKV12-41_AJ235953_Ig_kappa_variable_12-41_12	3	ILMN_1230194	619960	Igkv12-41	immunoglobulin kappa chain variable 12-41
IGKV12-44_AJ235955_Ig_kappa_variable_12-44_18	4	ILMN_1226667	619960	Igkv12-41	immunoglobulin kappa chain variable 12-41
IGKV12-46_AJ235956_Ig_kappa_variable_12-46_18	5	ILMN_1258548	692245	Igkv12-46	immunoglobulin kappa chain variable 12-46
IGKV4-54_AJ231223_Ig_kappa_variable_4-54_15	6	ILMN_2608768	385291	Igkv4-54	immunoglobulin kappa chain variable 4-54
scl28918.2.12_106	7	ILMN_1254159	16120	Igk-V38	immunoglobulin kappa chain variable 38(V38)
IGHV1S63_L26853_Ig_heavy_variable_1S63_4	8	ILMN_1225733	677643	LOC677643	similar to monoclonal antibody BBK-2 heavy chain (LOC677643), misc RNA.
IGHV1S36_M13788_Ig_heavy_variable_1S36_40	9	ILMN_2437019	---	IGHV1S36_M13788_Ig_heavy_variable_1S36_40	---
IGHV1S120_AF025443_Ig_heavy_variable_1S120_8	10	ILMN_2470564	16049	IGHV1S120	immunoglobulin alpha heavy chain variable region
scl0380794.2_30	11	ILMN_1249975	380794	Ighg	Immunoglobulin heavy chain (gamma polypeptide), transcript variant 1 (Ighg), mRNA.
IGHV14S1_X03571$M12813_Ig_heavy_variable_14S1_9	12	ILMN_2597469	544904	LOC544904	similar to idiotypic anti-NP IgG(1) heavy chain V-D-J (LOC544904), mRNA.
IGKV16-104_AJ235936_Ig_kappa_variable_16-104_171	13	ILMN_2515256	100046546	LOC100046546	similar to IgM (kappa)light-chain
IGKV6-14_Y15975_Ig_kappa_variable_6-14_11	14	ILMN_2699222	669053	LOC669053	similar to Ig kappa chain V-V region MPC11 precursor (LOC669053), mRNA.
IGKV6-25_AJ235962_Ig_kappa_variable_6-25_13	15	ILMN_2465985	676136	LOC676136	similar to Ig kappa chain V-V region MPC11 precursor (LOC676136), mRNA.

scl012228.1_44	16	ILMN_2685011	12228	Btg3	B-cell translocation gene 3
IGHV1S124_AF025449_Ig_heavy_variable_1S124_11	17	ILMN_2422974	16061	IGHV1S124	immunoglobulin heavy variable region
IGKV8-31_AJ235957_Ig_kappa_variable_8-31_3	18	ILMN_1230332	546905	Igkv8-31	similar to anti-DNA immunoglobulin light chain variable IgM
IGKV10-95_AF029261_Ig_kappa_variable_10-95_20	19	ILMN_2766296	434031	predicted gene 5573	predicted gene, EG434031, Igkv10-95
scl27608.15.10_44	20	ILMN_1245472	11576	Afp	alpha fetoprotein
IGKV4-68_AJ231222_Ig_kappa_variable_4-68_18	21	ILMN_2490758	384515	Gm1419	gene model 1419, (NCBI) IGKV4-68
GI_6678306	22	ILMN_2830821	21784	Tff1	trefoil factor 1
IGKV4-77_AJ235940_Ig_kappa_variable_4-77_18	23	ILMN_1258680	672339	LOC672339	similar to Ig kappa chain V-IV region S107B precursor (LOC672339), mRNA.
IGKV19-93_AJ235935_Ig_kappa_variable_19-93_104	24	ILMN_1238932	16120	Igk-V38	immunoglobulin kappa chain variable 38(V38)
scl40238.3.1_75	25	ILMN_2649275	259301	Leap2	liver-expressed antimicrobial peptide 2
scl0258722.1_9	26	ILMN_1253092	258722	Olfr611	olfactory receptor 611
IGKV4-61_AJ231209_Ig_kappa_variable_4-61_12	27	ILMN_2469743	236047	Igkv4-74	immunoglobulin kappa chain variable 4-74
IGKV4-52_AJ239198_Ig_kappa_variable_4-52_18	28	ILMN_2431017	723991	Igkv4-52	immunoglobulin kappa chain variable 4-52
IGKV6-15_Y15976_Ig_kappa_variable_6-15_15	29	ILMN_1222500	100047162	LOC100047162	similar to immunoglobulin kappa-chain (LOC100047162), mRNA.
IGKV1-88_AJ231206_Ig_kappa_variable_1-88_289	30	ILMN_2495896	692154	Igkv1-88	immunoglobulin kappa chain variable 1-88
scl16414.9.494_144	31	ILMN_1236315	70750	Kdsr	3-ketodihydrosphingosine reductase
IGKV4-73_AJ231216_Ig_kappa_variable_4-73_18	32	ILMN_1242258	628516	Gm6889	predicted gene, EG628516, Igkv4-73

scl47286.9_3	33	ILMN_1243014	68036	Zfp706	zinc finger protein 706
scl00025.1_14	34	ILMN_2738739	57752	Tacc2	transforming, acidic coiled-coil containing protein 2
scl54436.7_325	35	ILMN_2692306	20937	Suv39h1	suppressor of variegation 3-9 homolog 1 (Drosophila)
IGKV8-30_AJ235948_Ig_kappa_variable_8-30_91	36	ILMN_2631763	384419	Igkv8-30	immunoglobulin light chain variable region
IGKV12-67_AJ235933_Ig_kappa_variable_12-67_27	37	ILMN_2426436	628551	Igkv12-67	immunoglobulin kappa chain variable 12-67
scl0381816.1_161	38	ILMN_2593944	381816	4922502D21Rik	RIKEN cDNA 4922502D21 gene
scl22872.7_240-S	39	ILMN_2723788	66471	Anp32e	acidic (leucine-rich) nuclear phosphoprotein 32 family, member E
scl49283.17_415	40	ILMN_1216742	22061	Trp63	transformation related protein 63
scl30502.9.1_2	41	ILMN_2673121	24058	Sigirr	single immunoglobulin and toll-interleukin 1 receptor (TIR) domain
scl54976.5.1_9	42	ILMN_1215810	68995	Mcts1	malignant T cell amplified sequence 1
scl54594.3.1_69	43	ILMN_2463395	22037	Trap1a	tumor rejection antigen P1A
scl52590.16_341	44	ILMN_2738893	226090	Ermp1	endoplasmic reticulum metallopeptidase 1
scl16356.5_24	45	ILMN_1239134	72585	Lypd1	Ly6/Plaur domain containing 1
IGKV4-57_AJ231221_Ig_kappa_variable_4-57_15-S	46	ILMN_2463573	236047	Igkv4-74	immunoglobulin kappa chain variable 4-74
scl011799.4_8	47	ILMN_2632712	11799	Birc5	baculoviral IAP repeat-containing 5
scl0017433.2_285	48	ILMN_1223628	17433	Mobp	myelin-associated oligodendrocytic basic protein
scl057778.24_5	49	ILMN_2603689	57778	Fmnl1	formin-like 1

scl39207.10.1_15	50	ILMN_1219679	66179	1110031I02Rik	RIKEN cDNA 1110031I02 gene
scl11520.1.1_262	51	ILMN_2495367	171258	V1ri7	vomeronasal 1 receptor, I7
scl9411.1.1_287	52	ILMN_2690884	258749	Olfr556	olfactory receptor 556
scl0117160.17_30	53	ILMN_2522155	117160	Ttyh2	tweety homolog 2 (Drosophila)
scl0258886.1_30	54	ILMN_2603395	258886	Olfr1299	olfactory receptor 1299
scl52739.10.1_4	55	ILMN_2422676	22786	Zp1	zona pellucida glycoprotein 1
scl00100929.2_2	56	ILMN_1229311	100929	Tyw1	tRNA-yW synthesizing protein 1 homolog (S. cerevisiae)
scl067248.2_33	57	ILMN_2711551	67248	Rpl39	ribosomal protein L39
IGKV4-55_AJ231225_Ig_kappa_variable_4-55_21	58	ILMN_1247185	100047316	LOC100047316	similar to anti-MOG Z12 variable light chain (LOC100047316), mRNA.
IGKV4-62_AJ231210_Ig_kappa_variable_4-62_17	59	ILMN_1220577	636752	LOC636752	similar to Ig kappa chain V-VI region NQ2-6.1 (LOC636752), mRNA.
GI_31982096-S	60	ILMN_3160326	19092	Prkg2	protein kinase, cGMP-dependent, type II
scl49065.9.1_50	61	ILMN_2651781	67841	Atg3	autophagy-related 3 (yeast)
scl23055.15.1_74	62	ILMN_1244226	53317	Plrg1	pleiotropic regulator 1, PRL1 homolog (Arabidopsis)
scl0056357.2_122	63	ILMN_2728379	56357	Ivd	isovaleryl coenzyme A dehydrogenase
scl38509.3.1_171	64	ILMN_1257581	16545	Kera	keratocan
scl000615.1_57	65	ILMN_2659503	12554	Cdh13	cadherin 13
scl41112.7.1_150	66	ILMN_1214957	73634	1700125H20Rik	RIKEN cDNA 1700125H20 gene
scl18868.1.64_1	67	ILMN_	68032	Tmem85	transmembrane protein 85

		2657163			
scl050916.6_54	68	ILMN_2739870	50916	Irx4	Iroquois related homeobox 4 (Drosophila)
IGHV14S2_X03572$L29396_Ig_heavy_variable_14S2_16	69	ILMN_2769904	630322	LOC630322	similar to Ig heavy chain V region B1-8/186-2 precursor
scl067702.1_56	70	ILMN_2692563	67702	Rnf149	ring finger protein 149
scl0020646.1_10	71	ILMN_2631979	84704	Snurf	SNRPN upstream reading frame
IGKV4-79_AJ231214_Ig_kappa_variable_4-79_9	72	ILMN_2745775	213684	Gm194	gene model 194, (NCBI) IGKV4-79
scl51014.5.1_34	73	ILMN_2764325	56716	Gbl	G protein beta subunit-like
scl23236.14.1_102	74	ILMN_2701779	214048	Larp2	La ribonucleoprotein domain family, member 2
scl35959.8.1_6	75	ILMN_2769525	15288	Hmbs	hydroxymethylbilane synthase
scl28232.31_105	76	ILMN_2660147	74741	5730419I09Rik	RIKEN cDNA 5730419I09 gene
scl0022774.2_273	77	ILMN_1213145	22774	Zic4	zinc finger protein of the cerebellum 4
scl0074570.2_105	78	ILMN_2744259	74570	Zkscan1	zinc finger with KRAB and SCAN domains 1
scl0013134.2_302	79	ILMN_2720339	13134	Dach1	dachshund 1 (Drosophila)
scl33543.7.1_97	80	ILMN_2710639	330820	4933402J07Rik	RIKEN cDNA 4933402J07 gene
scl18443.13.1_19	81	ILMN_2648867	18041	Nfs1	nitrogen fixation gene 1 (S. cerevisiae)
IGKV6-23_AJ235961_Ig_kappa_variable_6-23_15	82	ILMN_2722893	637227	LOC637227	similar to Ig kappa chain V-V region MPC11 precursor (LOC637227), mRNA.
scl45352.5_13	83	ILMN_1230721	67065	Polr3d	polymerase (RNA) III (DNA directed) polypeptide D
scl24618.9_190	84	ILMN_12425	320541	A530082C11Rik	RIKEN cDNA A530082C11 gene

		42			
scl018194.8_27	85	ILMN_2609273	18194	Nsdhl	NAD(P) dependent steroid dehydrogenase-like
scl00108058.2_28	86	ILMN_1222123	108058	Camk2d	calcium/calmodulin-dependent protein kinase II, delta
scl0012848.2_316	87	ILMN_2740523	12848	Cops2	COP9 (constitutive photomorphogenic) homolog, subunit 2 (Arabidopsis thaliana)
scl37747.5_571	88	ILMN_2709001	216152	BC005764	cDNA sequence BC005764 (BC005764), mRNA
scl0320162.19_2	89	ILMN_1246221	320162	Ccdc45	coiled-coil domain containing 45
IGKV14-100_AJ231243_Ig_kappa_variable_14-100_12	90	ILMN_1232444	243439	Igkv14-100	immunoglobulin kappa chain variable 14-100
IGKV15-103_AJ231269_Ig_kappa_variable_15-103_262	91	ILMN_2483493	692169	IGKV15-103	immunoglobulin kappa chain variable 15-103
scl45509.5.1_248	92	ILMN_2653555	14939	Gzmb	granzyme B
scl54390.1.1_5	93	ILMN_1226793	260300	Gm1549	gene model 1549, (NCBI)
scl0018143.2_289	94	ILMN_2668835	18143	Npas2	neuronal PAS domain protein 2
scl34335.3_203	95	ILMN_2774681	26887	Chst4	carbohydrate (chondroitin 6/keratan) sulfotransferase 4
scl54914.7.1_116	96	ILMN_2510905	170828	Vgll1	vestigial like 1 homolog (Drosophila)
scl0001193.1_40	97	ILMN_2659006	269823	Pon3	paraoxonase 3
IGKV4-72_AJ231219_Ig_kappa_variable_4-72_18	98	ILMN_1243197	385109	Gm1499	gene model 1499, (NCBI), IGKV4-72
scl0014857.1_108	99	ILMN_2657636	14857	Gsta1	glutathione S-transferase, alpha 1 (Ya)
IGHV9S6_L14366_Ig_heavy_variable_9S6_92	100	ILMN_2478220	674147	LOC674147	similar to Ig heavy chain V-I region HG3 precursor

scl46285.11.1_214	101	ILMN_2639136	71412	Dhrs2	dehydrogenase/reductase member 2 (Dhrs2), mRNA.
scl000477.1_15	102	ILMN_2686087	66388	Cutc	cutC copper transporter homolog (E.coli)
scl0067628.2_320	103	ILMN_1215011	67628	Anp32b	acidic (leucine-rich) nuclear phosphoprotein 32 family, member B
scl4527.1.1_323	104	ILMN_1237204	258952	Olfr341	olfactory receptor 341
TRAV12D-1_X06308_T_cell_receptor_alpha_variable_12D-1_143	105	ILMN_2472831	630086	Trav12-1	T cell receptor alpha variable 12-1
scl32646.8.1_89	106	ILMN_1248952	16833	Ldhc	lactate dehydrogenase C
scl0241516.2_316	107	ILMN_2735121	241516	Fsip2	fibrous sheath-interacting protein 2
scl53440.6.1_16	108	ILMN_2591342	225884	BC021614	cDNA sequence BC021614
scl0068045.1_0	109	ILMN_1246483	68045	2700060E02Rik	RIKEN cDNA 2700060E02 gene
scl27604.2_9	110	ILMN_1230278	20311	Cxcl5	chemokine (C-X-C motif) ligand 5
scl21086.3.1_42	111	ILMN_2666764	78617	Cstad	CSA-conditional, T cell activation-dependent protein
scl23881.4_371	112	ILMN_2671878	73172	Dem1	defects in morphology 1 homolog (S. cerevisiae)
scl000319.1_7	113	ILMN_2755195	56215	Acin1	apoptotic chromatin condensation inducer 1
scl21127.13.3_15	114	ILMN_2689119	19730	Ralgds	ral guanine nucleotide dissociation stimulator
scl0108653.1_104	115	ILMN_2607434	108653	4933426K21Rik	RIKEN cDNA 4933426K21 gene
scl0003571.1_24	116	ILMN_2621122	109161	Ube2q2	ubiquitin-conjugating enzyme E2Q (putative) 2
scl0107993.3_117	117	ILMN_2731774	107993	Bfsp2	beaded filament structural protein 2, phakinin

scl000110.1_1	118	ILMN_1249264	18631	Pex11a	peroxisomal biogenesis factor 11a
IGKV8-18_Y15979_Ig_kappa_variable_8-18_12	119	ILMN_1255400	384419	Gm10883	predicted gene, Igkv8-30
scl28280.10.1_35	120	ILMN_2688894	66857	1100001H23Rik	RIKEN cDNA 1100001H23 gene
scl0002706.1_4	121	ILMN_1256890	70122	Mllt3	myeloid/lymphoid or mixed-lineage leukemia (trithorax homolog, Drosophila); translocated to, 3
scl0071770.1_48	122	ILMN_2711007	71770	Ap2b1	adaptor-related protein complex 2, beta 1 subunit
scl056078.2_10	123	ILMN_2670368	56078	Car5b	carbonic anhydrase 5b, mitochondrial
scl29002.2.1_9	124	ILMN_2772762	15405	Hoxa9	homeo box A9
scl23468.6.1_93	125	ILMN_1220280	110326	Tas1r1	taste receptor, type 1, member 1
scl41468.13.1_27	126	ILMN_1253178	11670	Aldh3a1	aldehyde dehydrogenase family 3, subfamily A1 (Aldh3a1), mRNA.
scl39249.20_276	127	ILMN_2729743	72055	Slc38a10	solute carrier family 38, member 10
IGHV1S28_X02460_Ig_heavy_variable_1S28_13	128	ILMN_2517595	111507	IGHV1S28	immunoglobulin heavy chain complex, mRNA
scl078833.3_67	129	ILMN_2589960	78833	Gins3	GINS complex subunit 3 (Psf3 homolog)
scl0387349.1_257	130	ILMN_2607229	387349	Tas2r121	taste receptor, type 2, member 121
scl30651.1_27	131	ILMN_1217590	20768	Sephs2	selenophosphate synthetase 2
scl4936.1.1_100	132	ILMN_2775910	258580	Olfr1023	olfactory receptor 1023
scl0001997.1_57	133	ILMN_2777405	13684	Eif4e	eukaryotic translation initiation factor 4E
IGHV3S2_M12435_Ig_heavy_variable_3S2_32	134	ILMN_2432255	630337	LOC630337	similar to Ig heavy chain V region 1B43 precursor (LOC630337), mRNA.
IGKV5-	135	ILMN_	381783	Igkv5-43	immunoglobulin kappa chain variable 5-43

43_AJ235973_Ig_kappa_variable_5-43_8		1251451			
scl51170.17.1_3-S	136	ILMN_1258674	321007	Serac1	serine active site containing 1
scl53375.12.1_13	137	ILMN_2771851	54525	Syt7	synaptotagmin VII
scl34866.14.1_23	138	ILMN_2692980	212326	Fam149a	family with sequence similarity 149, member A
scl022337.1_17	139	ILMN_2498695	22337	Vdr	vitamin D receptor
IGKV17-121_AJ231258_Ig_kappa_variable_17-121_16	140	ILMN_2612346	383196	LOC383196	hypothetical LOC383196 (LOC383196), mRNA.
scl22353.18_6	141	ILMN_1229418	12807	Hps3	Hermansky-Pudlak syndrome 3 homolog (human)
scl0020868.1_54	142	ILMN_2703341	20868	Stk10	serine/threonine kinase 10
scl0001103.1_81	143	ILMN_2629737	387314	Tmtc1	transmembrane and tetratricopeptide repeat containing 1
scl0067374.1_279	144	ILMN_1227559	67374	Jam2	junction adhesion molecule 2
scl45278.7.1_11	145	ILMN_2755998	66148	Dnajc15	DnaJ (Hsp40) homolog, subfamily C, member 15
scl0002546.1_0	146	ILMN_2701617	14573	Gdnf	glial cell line derived neurotrophic factor
scl16240.4.1_0	147	ILMN_2755281	329253	BC115961	hypothetical protein 6530439I21
IGKC_V00807_Ig_kappa_constant_192	148	ILMN_2704562	100047628	LOC100047628	similar to Chain L, Structural Basis Of Antigen Mimicry In A Clinically Relevant Melanoma Antigen System, transcript variant 3 (LOC100047628), mRNA.
scl4934.1.1_214	149	ILMN_2641178	258577	Olfr1026	olfactory receptor 1026
scl37650.12_395	150	ILMN_2669172	75317	4930547N16Rik	RIKEN cDNA 4930547N16 gene
scl0022218.1_0	151	ILMN_2632942	22218	Sumo1	SMT3 suppressor of mif two 3 homolog 1 (yeast)

scl0014269.2_161	152	ILMN_2723708	14269	Fnbp1	formin binding protein 1
scl53490.4_84	153	ILMN_1247676	73703	Cst6	cystatin E/M
scl068135.3_22	154	ILMN_2704585	68135	Eif3h	eukaryotic translation initiation factor 3, subunit H
scl39522.14_3	155	ILMN_2595163	50997	Mpp2	membrane protein, palmitoylated 2 (MAGUK p55 subfamily member 2)
scl38846.4.1_6	156	ILMN_1229993	30045	Dnajc12	DnaJ (Hsp40) homolog, subfamily C, member 12
scl016579.20_85	157	ILMN_2654532	16579	Kifap3	kinesin-associated protein 3
scl20359.10_330	158	ILMN_1243525	18457	Pldn	pallidin
scl35378.12_17	159	ILMN_2775360	102626	Mapkapk3	mitogen-activated protein kinase-activated protein kinase 3
scl51704.9.1_177	160	ILMN_1239483	246102	Rttn	rotatin
scl015357.2_29	161	ILMN_1225718	15357	Hmgcr	3-hydroxy-3-methylglutaryl-Coenzyme A reductase
scl30652.3_263	162	ILMN_1228334	66422	2410015N17Rik	RIKEN cDNA 2410015N17 gene
IGKV4-71_AJ231218_Ig_kappa_variable_4-71_20	163	ILMN_2513393	434586	Igkv4-71	immunoglobulin kappa chain variable 4-71
scl46658.31_139	164	ILMN_2670172	16402	Itga5	integrin alpha 5 (fibronectin receptor alpha)
scl38446.16_40	165	ILMN_1253131	53605	Nap1l1	nucleosome assembly protein 1-like 1
scl31073.10_61	166	ILMN_1243030	65098	Zfand6	zinc finger, AN1-type domain 6
scl079560.1_5	167	ILMN_2675587	79560	Ublcp1	ubiquitin-like domain containing CTD phosphatase 1
scl0019820.1_259	168	ILMN_2684450	19820	Rnf12	ring finger protein 12
scl0230597.3_20	169	ILMN_	230597	Zfyve9	zinc finger, FYVE domain containing 9

		2502525			
scl30914.6_156	170	ILMN_1255828	244180	E030002O03Rik	RIKEN cDNA E030002O03 gene
scl40581.3.1_34	171	ILMN_1243257	327860	OTTMUSG00000005065	predicted gene, OTTMUSG00000005065
scl36437.4.1700_28-S	172	ILMN_2732634	29873	Cspg5	chondroitin sulfate proteoglycan 5
scl37121.13.1_3	173	ILMN_2643495	235180	Fez1	fasciculation and elongation protein zeta 1 (zygin I)
scl0016071.1_62	174	ILMN_2684371	100047628	LOC100047628	similar to Chain L, Structural Basis Of Antigen Mimicry In A Clinically Relevant Melanoma Antigen System, transcript variant 3 (LOC100047628), mRNA.
scl013627.1_210	175	ILMN_2728756	13627	Eef1a1	eukaryotic translation elongation factor 1 alpha 1
scl41457.2_174	176	ILMN_1244424	11541	Adora2b	adenosine A2b receptor
scl0252903.3_21	177	ILMN_1223884	252903	Ap1s3	adaptor-related protein complex AP-1, sigma 3
scl0014580.2_305	178	ILMN_1214715	14580	Gfap	glial fibrillary acidic protein
scl22202.5_66	179	ILMN_2729113	73173	Pcdh18	protocadherin 18
scl19559.5.1_2	180	ILMN_2660461	77701	Lcn12	lipocalin 12
scl00103694.2_35	181	ILMN_2698606	103694	Tmed4	transmembrane emp24 protein transport domain containing 4
scl0066970.1_29	182	ILMN_1252746	66970	Ssbp2	single-stranded DNA binding protein 2
scl31573.3_3	183	ILMN_2777706	24030	Mrps12	mitochondrial ribosomal protein S12
scl55051.5.1_194	184	ILMN_1221225	236285	Lancl3	LanC lantibiotic synthetase component C-like 3 (bacterial)
scl25783.3.1_76	185	ILMN_2497349	67235	Zkscan14	zinc finger with KRAB and SCAN domains 14
scl0100715.1_21	186	ILMN_	100715	Papd4	PAP associated domain containing 4

		2629146			
scl49664.19_17	187	ILMN_2590219	268970	Arhgap28	Rho GTPase activating protein 28
scl0319772.2_40	188	ILMN_2676524	319772	C130050O18Rik	RIKEN cDNA C130050O18 gene
scl49758.2.1_108	189	ILMN_2655498	18188	Nrtn	neurturin
scl0001329.1_110	190	ILMN_1232420	55978	Ift20	intraflagellar transport 20 homolog (Chlamydomonas)
scl0002027.1_43	191	ILMN_2595115	56376	Pdlim5	PDZ and LIM domain 5
GI_84370245-I	192	ILMN_3162785	72780	Rspo3	R-spondin 3 homolog (Xenopus laevis)
scl23771.9.1_58	193	ILMN_2594881	54709	Eif3i	eukaryotic translation initiation factor 3, subunit I
scl8638.1.1_202	194	ILMN_1224906	171228	V1re5	vomeronasal 1 receptor, E5
scl30036.21_295	195	ILMN_2591414	52440	Tax1bp1	Tax1 (human T-cell leukemia virus type I) binding protein 1
scl39817.6_54	196	ILMN_1244358	20305	Ccl6	chemokine (C-C motif) ligand 6
scl4190.1.1_196	197	ILMN_2724923	258441	Olfr1310	olfactory receptor 1310
scl0003609.1_104	198	ILMN_1219747	26968	Islr	immunoglobulin superfamily containing leucine-rich repeat
scl0394435.7_126	199	ILMN_1222543	394430	Ugt1a10	UDP glycosyltransferase 1 family, polypeptide A10 (cluster)
scl0011799.1_42	200	ILMN_2603699	11799	Birc5	baculoviral IAP repeat-containing 5

Rank—Similarity to exemplar in Kmeans cluster profile

Gray—Immunoglobulin family genes
