# Supplementary material for: Critical Transition in Tissue Homeostasis Accompanies Murine Lung Senescence
Source: PLoS One. 2011 Jun 21;6(6):e20712. doi: 10.1371/journal.pone.0020712 (PMC3119663; doi:10.1371/journal.pone.0020712)
Supplement: Table S2 — Real-time PCR validation of genes in airspace peak. (PDF) [file pone.0020712.s007.pdf]

Supplemental Table 2: Fold change by Real-Time PCR of Selected Peak Genes[1]

Genes	4 vs 8-FC[2]	p Value	4 vs 12-FC[2]	p Value	8 vs 12-FC[2]	P Value
ATG3	1.21	0.03*	1.03	0.14	1.48	0.002**
GSTA2	0.91	0.37	0.91	0.22	3.85	0.002**
CDHI3	1.20	0.24	0.98	0.46	0.68	0.002**
LEAP2	1.50	0.006**	1.83	0.005**	0.56	0.07†
PRKG2	1.22	0.19	1.25	0.18	0.88	0.065†
DACH1	1.42	0.003**	1.98	0.07†	0.71	0.002**
GZMB	2.25	0.07†	3.0	0.007*	0.75	0.065†
TFF1	1.33	.93	0.903	0.343	2.67	0.05*

***p-<.05, **p-<.01, †p<.10**

[1]3-4 mice per time point

[2]FC-Fold Change
